# The effects of summer temperature, age and socioeconomic circumstance on Acute Myocardial Infarction admissions in Melbourne, Australia

**DOI:** 10.1186/1476-072X-9-41

**Published:** 2010-08-11

**Authors:** Margaret E Loughnan, Neville Nicholls, Nigel J Tapper

**Affiliations:** 1 Monash Climate, School of Geography and Environmental Science, Monash University, Wellington road, Clayton 3800, Australia

## Abstract

**Background:**

Published literature detailing the effects of heatwaves on human health is readily available. However literature describing the effects of heat on morbidity is less plentiful, as is research describing events in the southern hemisphere and Australia in particular. To identify susceptible populations and direct public health responses research must move beyond description of the temperature morbidity relationship to include social and spatial risk factors. This paper presents a spatial and socio-demographic picture of the effects of hot weather on persons admitted to hospital with acute myocardial infarction (AMI) in Melbourne.

**Results:**

In this study, the use of a spatial and socio-economic perspective has identified two groups within the population that have an increased 'risk' of AMI admissions to hospital during hot weather. AMI increases during hot weather were only identified in the most disadvantaged and the least disadvantaged areas. Districts with higher AMI admissions rates during hot weather also had larger proportions of older residents. Age provided some explanation for the spatial distribution of AMI admissions on single hot days whereas socio-economic circumstance did not. During short periods (3-days) of hot weather, age explained the spatial distribution of AMI admissions slightly better than socioeconomic circumstance.

**Conclusions:**

This study has demonstrated that both age and socioeconomic inequality contribute to AMI admissions to hospital in Melbourne during hot weather. By using socioeconomic circumstance to define quintiles, differences in AMI admissions were quantified and demographic differences in AMI admissions were described. Including disease specificity into climate-health research methods is necessary to identify climate-sensitive diseases and highlight the burden of climate-sensitive disease in the community. Cardiac disease is a major cause of death and disability and identifying cardiac-specific climate thresholds and the spatio-demographic characteristics of vulnerable groups within populations is an important step towards preventative health care by informing public health officials and providing a guide for an early heat-health warning system. This information is especially important under current climatic conditions and for assessing the future impact of climate change.

## Introduction

In recent years, several meteorological models have been developed to assess the impact of climate change on human health, in particular the effects of heatwave episodes [1-3]. Most of the published literature relates to mortality and to northern hemisphere climates [4-9]. Southern hemisphere regions (including Australia) by comparison have been less well represented in the literature [10-14]. Extending the studies to include Australia and morbidity is an important step in addressing the issue of climate change and health. Although there are some studies describing social vulnerability and mortality-heat relationships [4,5,15], there is, generally, a shortage of published research describing the relationships between morbidity, environmental exposure to heat, and social vulnerability. The identification of socioeconomic risk factors potentially contributing to heat related mortality and morbidity is crucial for the development and implementation of public health prevention and response. In this study we examine how spatial variations in social and demographic variables across a city (Melbourne, Australia) affect the health response to temperatures above certain thresholds. Many studies relating health and temperature have identified a "U" or "J" shaped temperature-mortality relationship, with a threshold temperature above which heat-related mortality and morbidity increase above baseline levels [16,17]. Threshold temperatures above which the hospital admission rate for acute myocardial infarction (AMI) increases for Melbourne Australia have been identified in a previous study [18]. Examining the spatial distribution of AMI admissions on days exceeding these threshold temperatures, as is done here, could provide information about the social and demographic characteristics of 'place' that may influence heat related morbidity.

This paper aims to address this problem by adding a socio-demographic perspective to the analysis of the effects of weather on persons with pre-existing cardiac disease within Melbourne. This research uses area-based socioeconomic indices in conjunction with area-based acute myocardial infarction (AMI) admission to hospital rates to highlight the effects of 'place' on population health.

## Materials and methods

Melbourne is Australia's second largest city with a resident population of approximately four million people. AMI was selected for this study as this is a serious cardiac condition which routinely results in hospitalisation; cardiovascular morbidity is the largest burden of disease in Australia [19]. AMI admissions were chosen rather than coronary deaths because using out-of-hospital death coding is more problematic than using information about hospital diagnosed cases such as non-fatal AMI. Non-fatal AMI events are of considerable medical, social, and economic importance, as they require medical interventions and considerably more resources than fatal AMI events. The data set used in this analysis consisted of all AMI admissions to hospitals in Melbourne during the period 1999-2004.

### Health data

The study population consisted of all subjects hospitalised in the 37 hospitals in the Statistical District (SD) of Melbourne, who were aged 35 years and older, who were resident in the SD of Melbourne, and had a principal diagnosis of AMI on admission. The 35 years and older cohort included 99.2% of all AMI admissions. The 55 years and older group consisted of 80.3% of these admissions and the 65 years and older group consisted of 61% of the admissions [20].

**Figure 1:**
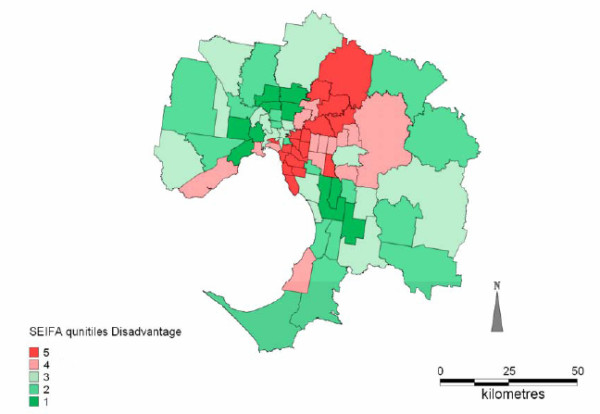
SEIFA (IRSD) quintiles for the Melbourne region (Quintile 1 most disadvantage, Quintile 5 least Disadvantage)

The International Classification of Disease version 10 codes I21.0 - I21.9 were used to define AMI. Data were supplied by the Victorian Department of Health (DoH) from the Victorian Admitted Episode Dataset (VAED) and included all five Melbourne hospital regions, age (in 5-year groups), sex, date of admission to hospital, principal diagnosis, and the Statistical Local Area (SLA) in which each patient resided. There are 75 SLAs in the Melbourne SD these are shown in Figure 1. The Monash University Standing Committee on Ethical Research involving Humans granted ethics approval for the study. These data have been previously used to demonstrate a seasonal difference in the spatial distribution of AMI admissions [20] and the threshold temperature for increased AMI admissions to hospital in Melbourne [18]. Demographic data for age (in 5-year groups) and sex (male and female) were obtained from the Australian Bureau of Statistics (ABS) as estimated mid-year population profiles for the SLA within the Melbourne SD aged 35 years and older [21]. AMI was expressed as age specific rates per million people within the population.

### Socioeconomic data

The measure of socioeconomic status used to examine health inequalities in this analysis was the 2001 Australian Bureau of Statistics (ABS) area-based Socio-Economic Index for Areas (SEIFA) [22]. This index is one of several constructed by the ABS to classify areas on the basis of social and economic information collected in the 2001 Census of Population and Housing. The SEIFA index for relative socioeconomic disadvantage (IRSD) was used in this analysis. SEIFA is derived from social and economic characteristics of the local area. A low SEIFA score would indicate characteristics such as low income, low educational attainment, and high levels of public housing, high unemployment, and jobs in relatively unskilled occupations. In this analysis, the SEIFA 2001 version was chosen as this is the approximate mid-point of our time series.

### Spatial data

Disease mapping itself is a way of visualizing complex relationships within the structure of the data, and revealing patterns of association. This study used statistical local area (SLA) data and as such displays the overall clustering tendency of AMI admissions to hospital in the Melbourne region. By using ecological analysis as the research design, and the assessment of association between disease incidence (AMI admissions) and environmental and social variables of interest, this research focuses on groups of individuals rather than individuals themselves. Demographic data for the number of person's aged 55 years and older per SLA were obtained from the ABS. The proportion of persons aged 55 years and older per SLA was calculated and mapped only to show where the older population live in Melbourne. All analyses were completed on the AMI cohort aged 35 years and older. The mapping tool used to integrate the health data with the geographical areas was MapInfo [23].

## Method

The SEIFA values for each SLA in Melbourne were divided into quintiles based on the Index of Relative Socioeconomic Disadvantage (IRSD) so that each quintile contained approximately 20% of the total Melbourne population 35 years and older (approximately 668,000 persons in each quintile). Each AMI admission to hospital was classified into a quintile according to the value of SEIFA for the SLA of usual residence. Quintile 1 includes the most disadvantaged SLAs and quintile 5 the least disadvantaged SLAs.

Only AMI admission data for the warmer months (November to March during 1999-2004) were used. Day of the week was not accounted for specifically because there was no statistically significant difference noted between the days of the week (in Melbourne admissions peak on Thursdays not Mondays as often reported elsewhere).

The threshold temperatures above which AMI admissions to hospital increase have previously been identified as 24-hour average (9 am to 9 am) temperature of 30°C, and a 3-day running mean of 24-hour average temperatures of 27°C [4]. However this method assumes population homogeneity in terms of demography and socioeconomic circumstance. How different subpopulations respond to hot weather is still unclear. To address this issue de-seasoned and de-trended AMI admissions and daily maximum, minimum, and mean temperatures were used to determine threshold temperatures for each socioeconomic quintile for both 24-hour average temperature and the 3-day running mean of 24-hour average temperature. This is the same method as described in Loughnan *et al *(2010). Seasonal decomposition procedures remove periodic fluctuations from time series data, such as annual or seasonal highs or lows. It is often used as a preliminary tool when analysing the trends in a time series data set. SPSS ver 14 was used for this analysis [24] and all other statistical analyses.

Box and whisker plots showing the median, inter-quartile points and range were produced to display the distribution of AMI admissions in both one and 3-degree Celsius bands for each of the socioeconomic quintiles separately. For daily maximum temperature, daily minimum temperature, and 24-hour average temperature (the average of the daily maximum and the daily minimum temperature between 9 am on one day and 9 am on the next day) were examined. The last of these incorporates the effects of a hot day followed by a hot night. To investigate the effect of consecutive days of hot weather a 3-day centred moving average of the 24-hour average temperature was calculated for the entire period 1999-2004. Three consecutive days was chosen as an averaging period because 3 days is the typical time taken for weather systems to move across the Melbourne region [25]. Maps of the spatial distribution of AMI admissions to hospital on days exceeding the thresholds were generated with MapInfo [23]. Demographic information relating to AMI admissions and their associated SEIFA quintiles were extracted from the November to March data set for days that exceeded the predetermined thresholds for hot weather (hot days) and days that did not exceed the threshold (all other summer days). Frequency distributions were generated for age and sex groups to determine the seasonal timing of events, and the percentage contribution of each age*sex group. Significance tests (*t-*tests) were used to determine significant differences between areas with AMI admissions on hot days and areas without AMI admissions on hot days. A General Linear Model multiple analysis of variance (MANOVA) was used to compare groups on a range of different characteristics, in this instance age and socio-economic circumstance. These analyses were done for both age and SEIFA (IRSD).

## Results

**Figure 2:**
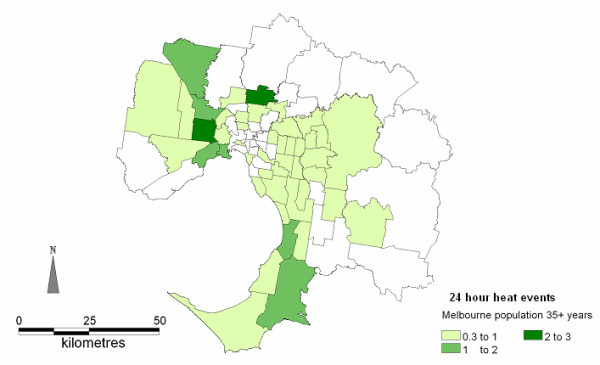
Spatial distributions of AMI admissions on days exceeding the 30°C threshold (AMI rates per million population)

**Figure 3:**
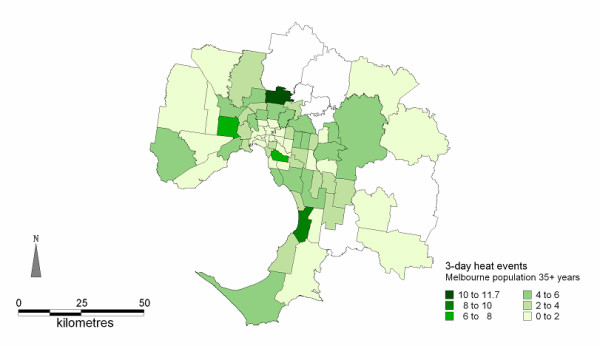
**Spatial distributions of AMI admissions on days exceeding the 27°C threshold (AMI rates per million population)**.

**Figure 4:**
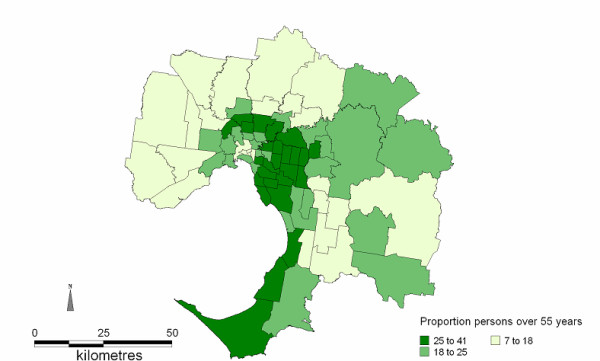
Proportion of persons aged 55 years and older in each SLA

Analyses of the AMI temperature relationships for each of the socioeconomic quintiles independently indicated that threshold temperatures above which AMI increased were only identified in the most disadvantaged and the least disadvantaged areas, i.e. quintile 1 and quintile 5 respectively. The results will first be presented for days exceeding the 30°C threshold, followed by the results for periods exceeding the 3-day average threshold of 27°C. Figure 1 shows a map of SEIFA (IRSD) quintiles for all 75 SLA. Maps detailing the spatial distribution of AMI on days exceeding the 30°C threshold and the 3-day threshold of 27°C were generated. These are shown in Figures 2 and 3 respectively. The proportion of older persons (55 years and older) in each SLA was also mapped; this is shown in Figure 4.

### Threshold temperature of 30°C

**Figure 5:**
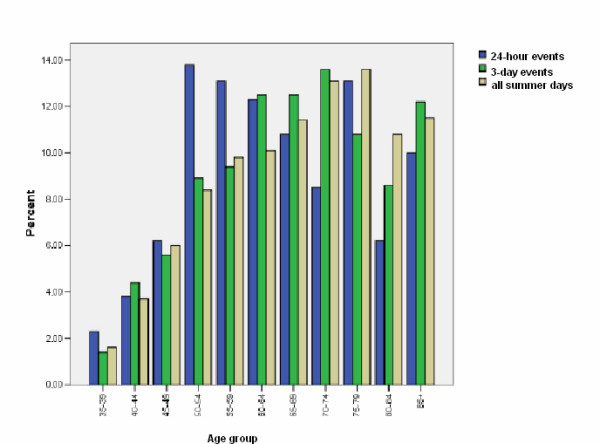
**Age distribution of AMI admissions during 24-hour and 3-day events and all days in summer**.

There were 130 people admitted to hospital with AMI on the 9 days that exceeded the temperature threshold during the study period. The average number of admissions on hot days was 14.6 admissions per day (95% CI 14.1-14.9). The daily average number of admissions during summer (November - March) was 13.1 (95% CI 12.7-13.5). This represents a 10.8% increase in AMI admissions, on days exceeding the threshold. Males and females admitted on days with temperatures exceeding 30°C were predominantly younger than 70 years of age. AMI admissions on all other days during the study period were generally older, as shown in Figure 5. There were more males than females (1.7:1) admitted [26]; this is consistent with the AMI admission population during summer but different to the general population of Melbourne where the sex ratio is close to 1:1. The males admitted on hot days were younger than males admitted on all other days in summer. The spatial distribution of AMI admissions on hot days shows higher rates in the north, north-east and southern suburbs of the Melbourne metropolitan area (see Figure 2). This is similar to the general spatial distribution of AMI in summer previously described for Melbourne [20]

Admissions on hot days came from 47 of the 75 SLAs in Melbourne. Independent samples *t*-tests were conducted to test whether or not there is a significant difference in the distribution of aged persons (% of the population aged 55 years and over) between the SLAs with AMI on hot days and the SLAs without AMI on hot days. The results indicated that there is a significant difference between the percentage of the population that is aged 55 years or over in SLAs with AMI on hot days (mean = 21.29%, SD = 5.91) and the percentage in SLAs without AMI on hot days (mean = 17.91%, SD = 5.2); *t *(73) = 2.568, p = 0.013). Similarly the difference in the SEIFA (IRSD) between the SLAs with AMI on hot days and the SLAs without AMI on hot days was tested. There was no significant difference between SEIFA (IRSD) in SLAs with AMI on hot days (mean = 1021, SD = 67) and SLAs without AMI on hot days (mean = 1043, SD = 53); *t *(73) = - 1.52, p = .133). A one-way between-groups multivariate analysis of variance (MANOVA) was performed to investigate the demographic differences in SLAs with and without AMI admissions to hospital on hot days. Two dependent variables were used: the percentage of persons aged 55 years and older on each SLA, and the SEIFA (IRSD) value of each SLA. The independent variable was a categorical variable (SLA without AMI admissions or SLA with AMI admissions). Preliminary assumption testing was conducted to check for normality, linearity, univariate and multivariate outliers with no serious violations noted. There was a statistically significant difference between SLAs without AMI admissions and SLAs with AMI admissions on the combined dependent variables; F (2, 72) = 4.71, p = 0.012; Wilks' Lambda = .884; partial eta squared = .116.

When the results for the dependent variables were considered separately, the only difference to reach statistical significance, using a Bonferroni adjusted alpha level of 0.02, was percentage of persons aged 55 years and over: F (1,74) = 6.44, p = 0.013, partial eta squared = .081.

### Threshold temperature of 3-day average 27°C

There were 14 episodes (41 days) when the 3-day average temperature was ≥ = 27°C; this resulted in 741 AMI admissions to hospital. This is an average of 18 admissions per day (95% CI 16.4-19.1). The daily average number of admissions during the warmer months (November-March) was 13.1 (95% CI 12.7-13.5). This represents a 37.7% increase in admissions during short episodes of heat. The distribution of AMI admissions over the 3 days was approximately equal with 31% of the total admissions on day 1, 34% of the total admissions on day 2, and 35% of the total admissions on day 3 [18]. Episodes exceeding the threshold predominately (73%) occurred in February. The spatial distribution of AMI admissions on consecutive hot days shows higher rates in the north, north-east and southern suburbs of the Melbourne metropolitan area and an escalation of the AMI rates in the eastern suburbs (see Figure 3).

Figure 5 indicates that more people aged between 55 and 74 years were affected during short periods of hot weather. Loughnan *et. al*. (2010) found that more males were affected(1.8:1) and the affected males were predominantly between 50 and 79 years of age, with the peak occurrence in the 60-64 years group. Females were in the main older than 70 years [18]. AMI admissions came from 67 of the 75 SLAs in Melbourne. The results again indicated that there is a significant difference between the percentages of persons aged 55 years and over in SLAs with AMI on 3 consecutive hot days (mean = 20.41, SD = 5.91) and SLAs without AMI on 3 consecutive hot days (mean = 15.28, SD = 2.21); *t *(73) = 4.64, p < 0.001). Levene's test for equality of variances was significant therefore equal variances were not assumed. There was also a significant difference in SEIFA (IRSD) in SLAs with AMI on 3 consecutive hot days (mean = 1025, SD = 62.5) and SLAs without AMI on 3 consecutive hot days (mean = 1078, SD = 40); *t *(73) = -2.197, p = .031). A MANOVA was performed to investigate the demographic differences in SLAs with and without AMI admissions to hospital on 3 consecutive hot days. There was a statistically significant difference between SLAs without AMI admissions and SLAs with AMI admissions on the combined dependent variables; F (2, 73) = 14.2, p < 0.001; Wilks' Lambda = .868; partial eta squared = .132. When the results for the dependent variables were considered separately, both age and SEIFA (IRSD) reached statistical significance, the percentage of persons aged 55 years and over: F (1,74) = 5.11, p = 0.02, partial eta squared = .065. SEIFA (IRSD) F (1,74) = 4.82, p = 0.03, partial eta squared = .062.

Both age and socioeconomic status contribute to the spatial distribution of AMI admissions in Melbourne during consecutive days of hot weather. Age provides a slightly better explanation of the spatial pattern of AMI admissions during hot weather than does socioeconomic status.

**Figure 6:**
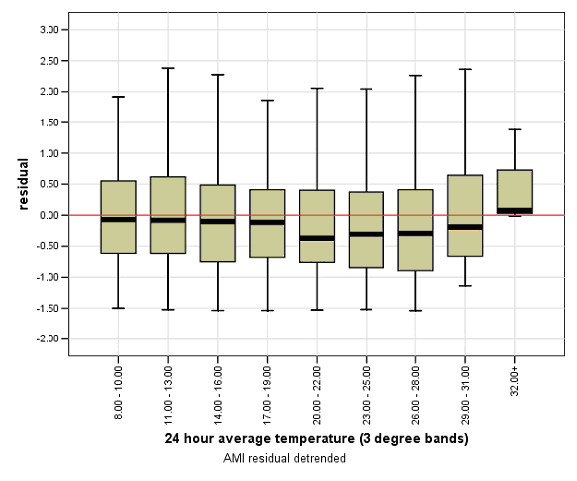
**Threshold temperatures for AMI admission from quintile 1 during 24-hour heat events**.

**Figure 7:**
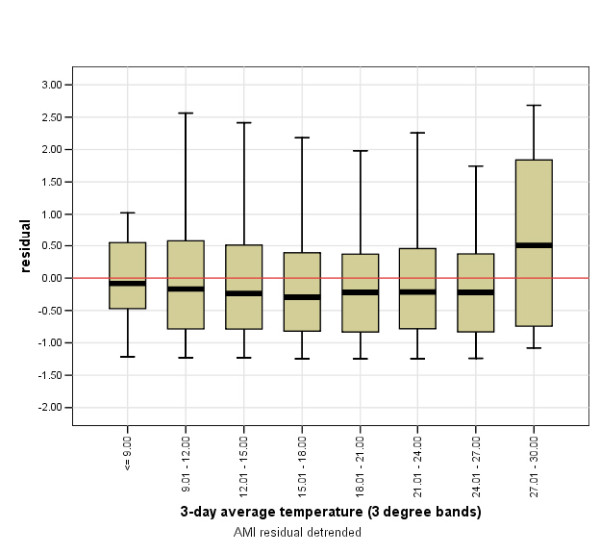
**Threshold temperatures for AMI admission from quintile 5 during 3-day heat events**.

An examination of the relationship between AMI admissions and temperature for each of the 5 socioeconomic quintiles indicated areas within quintile 1 (greater disadvantage) demonstrated increased numbers of AMI admissions on days exceeding the 32°C threshold (see Figure 6). However, quintile 5 (least disadvantage) exhibited a threshold above which AMI admissions increased when the 3-day average temperature was ≥ = 27°C (Figure 7).

## Discussion

Including socio-demographic and spatial information into analyses of AMI admissions and ambient temperature presents a more holistic picture of public health vulnerability to hot weather. In this study, the use of a spatial and socio-economic perspective has identified two groups within the population that have an increased 'risk' of AMI admissions to hospital during hot weather. Areas with higher AMI admissions rates during hot weather also had larger proportions of older residents. The contribution of age and socioeconomic circumstance to the previously described thresholds for Melbourne [18] indicates that the populations driving the thresholds appear to consist of residents from areas with the highest or lowest socio-economic standing.

The age demographic in quintile 1 (most disadvantaged) is younger than the other quintiles and the age of persons admitted to hospital with an AMI is correspondingly slightly younger. However, advancing age provided some explanation for the spatial distribution of AMI admissions on single hot days whereas socio-economic circumstance did not. Possible explanations for this are; many elderly pensioners live in poorer areas, persons living in areas of greater disadvantage are also more likely to have a greater number of co-morbidity and cardiac risk factors [27] thus, the quintile 1 population may have a higher cardiac risk. Quintile 1 also has a larger proportion of unskilled workers who may be at a greater risk of occupational exposure to heat. There are increased numbers of admissions amongst persons aged 50-64 years on days exceeding the 30°C threshold; potentially this could represent environmental exposure in an ageing workforce that has a higher risk of cardiac disease. This group may therefore respond to the initial impact of 'hot' weather. This data set only details place of residence, not place of occupation, nor the place where the person had their infarct. Therefore, it can only be suggested that unskilled workers may risk greater occupational/environmental exposure to heat. Further research at a finer spatial scale is required to clarify this association.

Conversely, quintile 5 (least disadvantaged) has an older population, especially older females. This age and sex demographic was described as the most vulnerable during the European heat wave in 2003 [28-30] this group was also acknowledged as high risk by the DoH following the Melbourne heatwave in 2009 [31]. The areas that comprise quintile 5 are located in the inner Melbourne region and may experience greater effects of the Melbourne heat island during consecutive 'hot' days. This effect may be enhanced by poor air quality when the increased temperature is accompanied by low wind speed.

During short periods of hot weather, age explained the spatial distribution of AMI admissions slightly better than socioeconomic circumstance. However the relationship between AMI admission and socio-economic circumstance during these short periods of hot weather was significant and negative. Therefore, it must also be considered that data at an SLA level has undergone considerable smoothing and individual or small area characteristics are masked. Small areas of greater disadvantage may be located within quintile 5 areas, such as areas of urban overcrowding in government housing and high rise apartments. The percentage contribution to AMI admissions from these areas cannot be determined from this data set, but should be considered in future research at a finer spatial scale.

The maps of the spatial distribution of AMI admissions on days exceeding both the predefined thresholds in Figures 2 and 3 indicate that as the duration of hot weather increased from 1 day to 3 days the number of affected SLAs expands from 47 SLAs to 67 SLAs, and the numbers of admissions from the south and south-eastern suburbs increased. This extension in AMI admissions is into areas with higher proportions of people aged 55 years and older (Figure 4).

The main limitations of this study were not being able to determine the amount of exposure experienced by people admitted with AMI on hot days, where they were and what level of activity they were engaged in. The spatial area of aggregation at an SLA level is still quite large and distinct 'hot spots' within these regions may exist. The analysis included single day's ≥ = 30°C; some of these days were potentially included in the 3-day analysis. Therefore, the results should be seen as complementing each other not as distinct entities. Future research should include analysis at a finer spatial resolution and an indication of potential occupational exposure to ambient heat. Another limitation of this study was not having information detailing co-morbidities of the persons admitted with AMI. Future research into AMI admissions and climate should consider including co-morbidities in a more comprehensive data set.

### Concluding remarks

Assessing the potential health impacts of weather requires two things: firstly determining the vulnerability of a specified population, and secondly assessing its ability to adapt and change in response to climatic stress. Including disease specificity into climate-health research methods is necessary to identify climate-sensitive diseases and highlight the burden of climate-sensitive disease in the community. For example, a measure of 'all cause' mortality in Australia includes a large proportion of coronary disease and AMI.

Simply because coronary artery disease is the most common cause of death and disability in Australia [19,27], at any given time there is a sizeable 'susceptible pool' of individuals likely to respond when exposed to weather extremes. This problem can be addressed by directing research to identify cardiac-specific climate thresholds and the spatio-demographic characteristics of vulnerable groups within populations, as was done in this study. This information can inform public health officials and provide a guide for an early heat-health warning system. Furthermore, disease specific research can provide information to help direct preventative strategies to reduce the incidence of coronary disease within communities, thereby reducing the population at risk during hot weather.

Health precedes disease and with respect to climate change, preserving health may help preclude or reduce heat related death or disability in the future.

Overall, the analysis has demonstrated that both age and socioeconomic inequality contribute to AMI admissions to hospital in Melbourne during hot weather. By using socioeconomic circumstance to define quintiles, differences in AMI admissions were quantified and demographic differences in AMI admissions were described. In addition, the response of each quintile to temperature thresholds was determined and explained.

During periods of hot weather AMI admissions were concentrated in SLA to the northwest, east and southern regions of the metropolitan area.

Increased rates of AMI persist in the northern and southern suburbs during both single and 3-day events. The AMI rate in the eastern suburbs increases as the duration of hot weather persists; suggesting that the persistent heat evokes vulnerability in a population with lower risk during single hot days. This information is especially important under current climatic conditions and for assessing the future impact of climate change. Climate change models for Australia suggest that south-eastern Australia is likely to experience a 0.2-1.4°C increase in temperature by 2050 and heat-waves will become more frequent [32]. The number of days with temperatures over 35°C are expected to increase by 9-12 days per year by 2030 [32]. Should this be the case temperature thresholds would be exceeded more frequently. In addition, if this included increased incidence of consecutive days of 'hot' weather the health related impact could be even more dramatic. Further research needs to undertaken within the identified 'at risk' groups to ascertain any possible social or environmental causes of AMI during periods of increased temperature.

## Competing interests

The authors declare that they have no competing interests.

## Authors' contributions

ML contributed to the initial study design, data acquisition, and analysis, interpretation of results and drafting of the maps and the manuscript. NN provided advice relating to analysis of data and interpretation of the results and revising the manuscript. NT contributed to the study design, and interpretation of results and revising the manuscript.

All authors have read and approved the final manuscript.
